# The Relationship Between Gut Microbiome *Bifidobacterium* and Anti-tumor Immune Responses in Esophageal Squamous Cell Carcinoma

**DOI:** 10.1245/s10434-024-16288-4

**Published:** 2025-03-04

**Authors:** Haolin Wang, Yoshifumi Baba, Yoshihiro Hara, Tasuku Toihata, Keisuke Kosumi, Kazuto Harada, Masaaki Iwatsuki, Yuji Miyamoto, Hideo Baba

**Affiliations:** Department of Gastroenterological Surgery, Graduate School of Medical Sciences, Kumamoto University, Kumamoto, Japan

**Keywords:** Gut microbiome, *Bifidobacterium*, Esophageal squamous cell carcinoma, Anti-tumor responses, Nutritional status

## Abstract

**Background:**

The *Bifidobacterium* genus is a prominent bacterial population in the gastrointestinal tract. Previous findings suggest that *Bifidobacterium* is linked to tumor suppression in mouse models of melanoma. Additionally, when combined with the programmed death-ligand 1 (PD-L1) antibody, it can enhance anti-tumor treatment by increasing tumor-specific T-cell responses and promoting infiltration of antigen-specific T cells into tumors. However, there is a lack of studies on *Bifidobacterium* in esophageal squamous cell carcinoma (ESCC). This study aimed to investigate the potential impact of *Bifidobacterium* on this cancer type.

**Methods:**

We examined 213 samples from ESCC patients who underwent tumor resection. The presence of *Bifidobacterium* was confirmed using quantitative polymerase chain reaction and fluorescent in situ hybridization (FISH). Patient overall survival (OS) was analyzed with *Bifidobacterium* positivity. Tumor-infiltrating lymphocytes (TILs) were evaluated via hematoxylin and eosin stains, and immunohistochemistry was used to assess programmed death-1 (PD-1), PD-L1, cluster of differentiation 8 (CD8), and forkhead box P3 (FOXP3) expression. Nutritional status was evaluated via computed tomography scans.

**Results:**

*Bifidobacterium* positivity showed no correlation with patient OS or TIL levels; however, *Bifidobacterium* positivity in normal tissue was associated with lower FOXP3 levels, suggesting a potential role in upregulating anti-tumor immune responses. Patients with *Bifidobacterium* present in peritumor normal tissue exhibited better skeletal muscle area and volume. Conversely, *Bifidobacterium* positivity in tumor tissue was associated with poorer prognostic nutrition index values, likely due to decreased albumin levels.

**Conclusion:**

*Bifidobacterium* can induce the upregulated anti-tumor immune response and is more prevalent in cases with good nutritional status.

**Supplementary Information:**

The online version contains supplementary material available at 10.1245/s10434-024-16288-4.

Esophageal cancer ranks seventh in incidence and sixth in mortality, contributing to approximately 1 in every 20 cancer-related deaths.^1^ In Asia, esophageal squamous cell carcinoma (ESCC) predominates, with a 5 year overall survival (OS) rate of 15–25% and a median OS of only 13 months.^1–3^ Endoscopic treatment is effective for early esophageal cancer,^4–6^ while locally advanced cases require multimodality therapy, including surgery with preoperative chemoradiotherapy (CRT) or perioperative chemotherapy (CT).^1,7^ Immune checkpoint inhibitors (ICIs) have emerged as first-line treatments for advanced esophageal cancer, showing efficacy, especially antibodies targeting programmed death-1 (PD-1) and programmed death-ligand 1 (PD-L1). However, only a minority of patients exhibit sustained responses, with resistance mechanisms classified into primary and acquired types.^8^

Factors affecting ICIs response include the microbiome, which plays crucial roles in human health, including tumor initiation and progression, by modulating the immune system.^9–12^ Recent research highlights the importance of the esophageal microbiome in overall health and its specific involvement in diseases such as Barrett’s esophagus and inflammatory conditions.^13^ *Bifidobacterium*, prevalent in the gastrointestinal tract, has been implicated in suppressing inflammatory diseases and influencing cancer development (Table 1). *Bifidobacterium* species influence gastrointestinal disease processes through various mechanisms and exhibit anti-tumor effects by modulating immune responses, such as inducing apoptosis in gastric cancer and suppressing colorectal carcinogenesis.^14–18^ These species enhance the anti-tumor immune response by altering the tumor microenvironment and influencing immune cell interactions. However, research on the presence of *Bifidobacterium* in ESCC tissues and its impact on anti-tumor immunity and patient survival is lacking. Its potential as a prognostic biomarker warrants further investigation.

**Table 1:** Effects of *Bifidobacterium* species on other gastrointestinal tract disease

Bacteria species	Disease	Models	Effects	Mechanisms	References
*B. breve*	Colorectal cancer	C57BL/6 mice	Inhibit colorectal tumorigenesis	*B. breve-*mediated tryptophan metabolism ameliorates the precancerous inflammatory intestinal milieu	Li et al.^14^
*B. lactis* BLa80	Inflammatory bowel disease	C57BL/6 J mice	Alleviate symptoms of intestinal inflammation	–	Dong et al.^15^
*B. adolescentis*	Colorectal cancer	BALB/c nude mice	Suppress colorectal carcinogenesis	Induce CD143+ CAFs with highly expressed GAS1 in the tumor microenvironment	Chen et al.^16^
*B. adolescentis*	Colorectal cancer	C57BL/6 wild-type mice	Suppress colorectal carcinogenesis	Induce Decorin+ macrophages	Lin et al.^17^
*B. bifidum*	Gastric cancer	BALB/c nude mice	Induce tumor apoptosis	Enhance Akt-p53-dependent apoptosis	Kim et al.^18^

*CAF* cancer-associated fibroblasts, *GAS1* growth arrest specific 1

This study examined 213 ESCC cases to investigate the presence of *Bifidobacterium* and its prognostic significance. Additionally, the study evaluated the relationship between anti-tumor immunity, *Bifidobacterium* presence, and patients’ preoperative nutritional status.

## Method

### Study Group

We analyzed 213 formalin-fixed paraffin-embedded (FFPE) ESCC tissue specimens from patients who underwent tumor resection at Kumamoto University Hospital in Japan from 24 October 2005 to 3 December 2018. All patients had histologically confirmed diagnoses. Pathologic diagnoses and clinicopathological factors were established based on the 7th Edition of the American Joint Committee on Cancer Staging Manual. Tumor staging was conducted accordingly. OS was defined as the time from the date of surgery to the date of death.

### DNA Extraction and Quantitative Polymerase Chain Reaction (qPCR) for Bifidobacterium Genomic

We extracted DNA for *Bifidobacterium* genus quantitative polymerase chain reaction (qPCR) from the FFPE esophageal cancer tissues using the QIAamp DNA FFPE Tissue Kit (Qiagen). qPCR was performed to measure the amount of DNA of the *Bifidobacterium* genus. Custom TaqMan primer/probe sets (Applied Biosystems) targeting the 16S ribosomal RNA gene DNA sequence of *Bifidobacterium* at the genus level and the reference gene, 16S, were utilized.^19^ Each reaction contained 100 ng of genomic DNA and was assayed in 10 μL reactions comprising 1× final concentration of LightCycler 480 Probe Master (Roche) and each TaqMan Gene Expression Assay (Applied Biosystems), in a 384-well optical PCR plate. Amplification and detection of DNA were performed with a LightCycler 480 Instrument II (Roche) under the following reaction conditions: 95 °C for 10 min, followed by 45 cycles of 15 s at 95 °C and 1 min at 60 °C. The primers and probes used were *Bifidobacterium* genus forward primer: 5′-CGGGTGAGTAATGCGTGACC-3′; *Bifidobacterium* genus reverse primer: 5′-TGATAGGACGCGACCCCA-3′; *Bifidobacterium* genus FAM probe: 5′-CTCCTGGAAACGGGTG-3′; SLCO2A1 forward primer: 5′-ATCCCCAAAGCACCTGGTTT-3′; SLCO2A1 reverse primer: 5′-AGAGGCCAAGATAGTCCTGGTAA-3′; and SLCO2A1 VIC probe: 5′-CCATCCATGTCCTCATCTC-3′. Each specimen was analyzed in duplicate for each target in a single batch, and the average cycle threshold (Ct) values for each target were used. The amount of *Bifidobacterium* genus in each specimen was calculated as a relative unitless value normalized with SLCO2A1 using the 2-ΔCt method (where ΔCt = the average Ct value of intestinal microbes—the average Ct value of SLCO2A1) as previously described.^19^ The amount of *Fusobacterium nucleatum* DNA was evaluated using the same method as in our previous paper.^20^

### Tumor-Infiltrating Lymphocyte (TIL) Evaluation, Immunohistochemistry, and Image Analysis

Hematoxylin and eosin (H&E)-stained tissue sections were evaluated for the presence and density of tumor-infiltrating lymphocytes (TILs), by a pathologist (YB) blinded to other data. Lymphocyte infiltration at the tumor invasive margin was graded as absent (score 0), mild (score 1), moderate (score 3), or strong (score 4). Expression of PD-1, PD-L1, cluster of differentiation 8 (CD8), and forkhead box P3 (FOXP3) was assessed using FFPE tissue samples sectioned at 4 μm. Following dewaxing and antigen retrieval, slides were treated with primary antibodies against CD8 (mouse monoclonal anti-human CD8 antibody, clone C8/144B, 1:100 dilution; Dako Cytomation), FOXP3 (mouse monoclonal anti-human FOXP3 antibody, clone 206D, 1:100 dilution; BioLegend), PD-1 (D4W2J, rabbit monoclonal antibody, 1:200 dilution; Cell Signaling Technology), and PD-L1 (clone E1L3N, 1:200 dilution; Cell Signaling Technology) overnight at 4 °C. Slides were then incubated with anti-rabbit or anti-mouse EnVision™+/horseradish peroxidase secondary antibody (Dako Japan) and visualized with 3,3-diaminobenzidine.

For technical reasons, not all data were available for each case as the subsequent analysis was performed only if three high-quality photos were represented. From the initial population of 213 patients in this study, pathological material after quality control was available and was successfully analyzed in 186, 207, 194, and 186 patients for PD-1, PD-L1, CD8, and FOXP3, respectively. PD-L1 expression on ESCC cells was assessed based on the percentage of stained cancer cells, categorized as no expression, or weak, moderate, or strong expression. PD-1-, CD8-, and FOXP3-positive lymphocytes were counted in three fields at the tumor invasive margin using a BZ-X700 digital microscope at 100× magnification (Keyence, Osaka, Japan) and hybrid cell count software (BZ-3HC; Keyence).

### Nutritional Status

We retrospectively calculated waist circumference, visceral fat area (cm^2^), subcutaneous fat area (cm^2^), skeletal muscle area, and skeletal muscle volume from the most recent preoperative CT images. Waist circumference, visceral fat area, and subcutaneous fat area were determined at the umbilicus level, with the cross-sectional area computed in cm^2^ using a window width of −190 to −30 HU. The skeletal muscle area was measured at the level of the third lumbar vertebra in the inferior direction, with the patient in the supine position. Muscle compartments were delineated using a window width of −30 to 150 HU, and their cross-sectional areas, in cm^2^ and volumes in cm^3^, were computed using the Volume Analyzer Synapse Vincent 3D image analysis system (Fujifilm Medical, Tokyo, Japan).

### Fluorescent In Situ Hybridization (FISH)

Fluorescent in situ hybridization (FISH) was employed to confirm the presence of *Bifidobacterium* in FFPE esophageal cancer specimens (4-μm-thick tissue sections). FFPE tissue specimens underwent deparaffinization using an automated system with passages through xylene (3 × 10 min), 100% alcohol (2 × 5 min), 95% ethanol (5 min), and, finally, 70% ethanol (5 min). The deparaffinized sections were then immersed in 2× standard saline citrate (SSC) for 5 min, microwaved in 2× SSC for 10 min, and cooled. Subsequently, the deparaffinized sections were incubated in a 0.02–0.5% pepsin/0.1N HCl solution at 37 °C for 30 min, washed in phosphate-buffered solution (PBS), dehydrated, and dried in an alcohol series. The Eub338 probe was utilized to identify total bacteria. A solution containing 10 µL of *Bifidobacterium* probe mixed with 0.5 µL of EUB338 probe was applied to the specimen and covered with parafilm or a cover glass. Hybridization was carried out overnight at 46 °C in a humidified box. After overnight incubation, the specimens were immersed in a wash solution at 46 °C for 20 min, followed by mounting after 4′,6-diamidino-2-phenylindole (DAPI) staining. *Bifidobacterium* was identified using a BZ-X700 digital microscope at a magnification of 200× (Keyence).

### Statistical Analysis

All data were processed and analyzed using JMP16 software (SAS Institute, Cary, NC, USA); statistical significance was defined as a *p*-value <0.05. Student’s t-test or a Chi-square analysis assessed the associations of *Bifidobacterium* presence within tumor or peritumor normal tissue with the following variables (as outcome variables): nutritional status (waist circumference, subcutaneous fat, visceral fat, skeletal muscle area, skeletal muscle volume, and prognostic nutrition index [PNI]), TILs, and the density of T cells in esophageal cancer tissue (PD1, PD-L1, FOXP3, and CD8 cells). The PNI is calculated using serum albumin levels and peripheral blood lymphocyte counts, serving as indicators of nutritional and immune states in cancer patients. Additionally, we analyzed the relationship between the amount of *Bifibacterium* and *Fusobacterium* in esophageal cancer tissues. The Kaplan–Meier analysis compared survival between patient groups and generated curves evaluating the OS of ESCC patients.

## Results

### FISH and qPCR for Detecting Bifidobacterium and Long-Term Outcomes

**Fig. 1:**
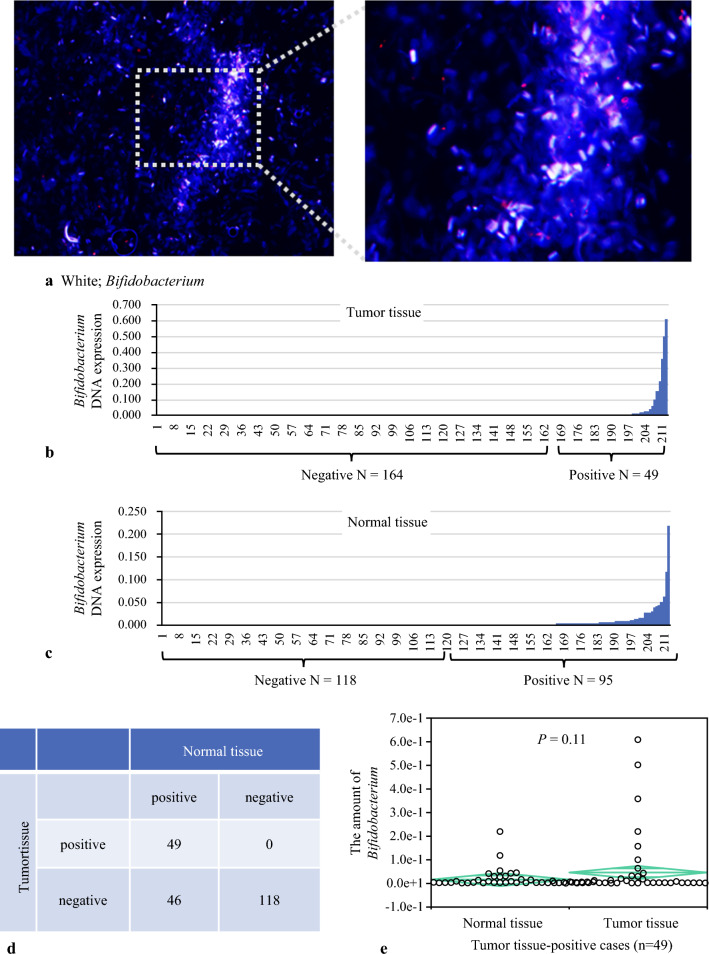
**a** FISH-stained sections of Bifidobacterium were hybridized with a Cy3-labeled probe Bif164, identified in FFPE tissue from ESCC patients. Original magnification, ×200. **b** 49/213 patients showed *Bifidobacterium* positivity in tumor tissue. **c** 95/213 patients showed *Bifidobacterium* positivity in normal tissue. **d** 49 patients showed *Bifidobacterium* was present in both tumor and normal tissue; 46 patients showed *Bifidobacterium* was only present in normal tissue; 118 patients showed absence of *Bifidobacterium* in both tumor and normal tissue. **e** Presence of *Bifidobacterium* in both tumor and normal tissue showed no significant difference in the amount of *Bifidobacterium* between tumor and normal tissue. *FISH* fluorescent in situ hybridization, *FFPE* formalin-fixed paraffin-embedded, *ESCC* esophageal squamous cell carcinoma

**Fig. 2:**
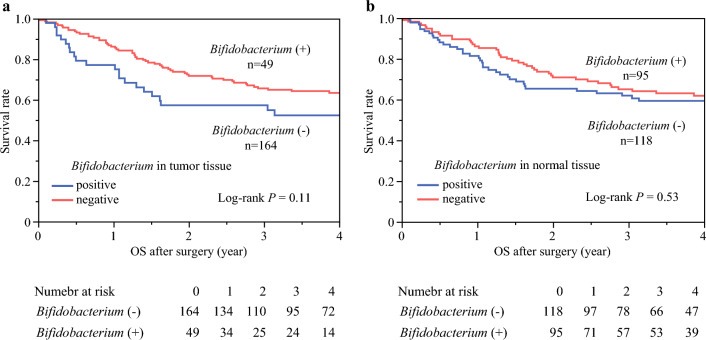
Kaplan–Meier analysis evaluating OS based on the presence of *Bifidobacterium* DNA in **a** tumor tissue and **b** normal tissue. *OS* overall survival

First, we confirmed the presence of *Bifidobacterium* in esophageal cancer tissue using FISH (Fig. 1a), and subsequently quantified *Bifidobacterium* levels in the FFPE samples of 213 incident ESCC cases using quantitative PCR assay. *Bifidobacterium* genus was detected and measured in both tumor and adjacent peritumor tissues of these 213 ESCC samples, with the levels depicted in Fig. 1b and c. The results showed that 49 (23%) tumor samples were *Bifidobacterium*-positive (Fig. 1b), while 95 (45%) peritumor samples were *Bifidobacterium*-positive (Fig. 1c). *Bifidobacteria* were found in both carcinomas and peritumor tissues in 49 pairs, exclusively in peritumor tissues in 46 cases, and absent in both tissues in 118 pairs (Fig. 1d). The 49 cases with *Bifidobacterium* in both tumor and normal tissues show no significant difference in the amount of *Bifidobacterium* (Fig. 1e). Relationships between the presence of *Bifidobacterium* and clinical and pathological features are summarized in Tables 2 and 3. The presence or absence of *Bifidobacterium* in tumor tissue and normal tissue did not show significant relevance to these pathological features. We previously reported that the oral bacterium *Fusobacterium nucleatum* influences the prognosis, CT resistance, and tumor immunity of esophageal cancer.^21–23^ There is no significant correlation between the amount of *Bifidobacterium* and *Fusobacterium* in esophageal cancer tissues. The data indicate that these two bacterium types do not show a relationship in this context. This finding is detailed in electronic supplementary material (ESM) Fig. S1a. Kaplan–Meier analysis was conducted to assess the association between *Bifidobacterium* and OS, with a median follow-up time of 7.1 years for all 213 patients. The presence of Bifidobacterium in neither tumor tissue nor normal tissue demonstrated a significant association with OS (Figs. 2a, b). These findings suggest that *Bifidobacterium* may not serve as a prognostic factor for OS in this patient cohort.

**Table 2:** Clinical, pathologic, and molecular characteristics, densities of T cells, or histologic lymphocytic reaction patterns of esophageal cancer cases according to the existence of *Bifidobacterium* DNA in esophageal cancer tumor tissue

Characteristic	All cases [*n* = 213]	Existence of *Bifidobacterium* in esophageal cancer tissue		*p*-Value
		Positive [*n* = 49]	Negative [*n* = 164]	
Sex				0.13
Female	31	4	27	
Male	182	45	137	
Mean age, years		68.5	67.2	0.38
Tobacco				0.71
Yes	179	42	137	
No	34	7	27	
EtOH				0.60
Yes	182	43	139	
No	31	6	25	
Grade				0.25
0	3	0	3	
1a	58	14	44	
1b	22	9	13	
2	4	1	3	
Tumor location				0.45
Upper	24	8	16	
Middle	85	18	67	
Lower	102	22	80	
pTNM stage				0.21
I	35	6	29	
II	61	16	45	
III	66	14	52	
IV	51	13	38	
Preoperative treatment				0.63
Presence	98	24	74	
Absence	115	25	90	
Performance status				0.96
0	181	41	140	
1	28	7	21	
2	4	1	3	
3	0	0	0	
4	0	0	0	
PD-1^+^ cell, mean		34.5	38.5	0.45
PD-L1^+^				0.83
Weak expression	184	45	139	
Moderate expression	9	2	7	
Moderately strong expression	6	1	5	
Strong expression	8	1	7	
CD8^+^ cell, mean		258.2	300.0	0.56
Tumor-infiltrating lymphocytes				0.02
Absent/mild	43	17	26	
Moderate	102	18	84	
Strong	53	12	41	

*EtOH* ethanol, *PD-1* programmed death-1, *PD-L1* programmed death-ligand 1

**Table 3:** Clinical, pathologic, and molecular characteristics, densities of T cells, or histologic lymphocytic reaction patterns of esophageal cancer cases according to the existence of *Bifidobacterium* DNA in esophageal cancer peritumor normal tissue

Characteristic	All cases [*n* = 213]	Existence of *Bifidobacterium* in peritumor tissue of esophageal cancer		*p*-Value
		Positive [*n* = 95]	Negative [*n* = 118]	
Sex				0.95
Female	31	14	17	
Male	182	81	101	
Mean age, years		67.2	67.7	0.70
Tobacco				0.29
Yes	179	77	102	
No	34	18	16	
EtOH				0.22
Yes	182	78	104	
No	31	17	14	
Grade				0.92
0	3	1	2	
1a	58	29	29	
1b	22	12	10	
2	4	2	2	
Tumor location				0.79
Upper	24	12	12	
Middle	85	36	49	
Lower	102	46	56	
pTNM stage				0.27
I	35	15	20	
II	61	31	30	
III	66	25	41	
IV	51	24	27	
Preoperative treatment				0.84
Presence	98	43	55	
Absence	115	52	63	
Performance status				0.44
0	181	79	102	
1	28	13	15	
2	4	3	1	
3	0	0	0	
4	0	0	0	
PD-1^+^ cell, mean		39.3	35.9	0.44
PD-L1^+^				0.28
Weak expression	184	82	102	
Moderate expression	9	4	5	
Moderately strong expression	6	5	1	
Strong expression	8	4	4	
CD8^+^ cell, mean		282.1	295.8	0.82
Tumor-infiltrating lymphocytes				0.17
Absent/mild	43	24	19	
Moderate	102	41	61	
Strong	53	27	26	

*EtOH* ethanol, *PD-1* programmed death-1, *PD-L1* programmed death-ligand 1

### TIL Distribution and Tumor Immune Response

**Fig. 3:**
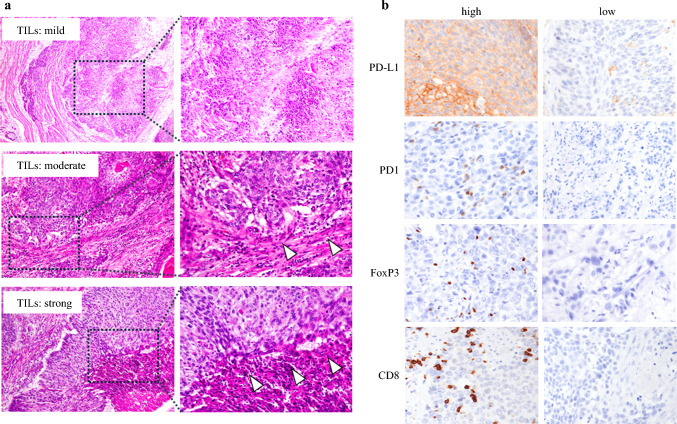
Anti-tumor response was evaluated through H&E staining and IHC using FFPE samples. **a** H&E staining sections of ESCC. TILs were assessed at tumor margin and categorized into four groups based on the degree of infiltration (absent, mild [upper], moderate [middle], strong [lower]). Original magnification, ×100. **b** Representative images of IHC staining for PD-L1 (upper), PD-1 (upper middle), FOXP3 (lower middle), and CD8 (lower). All cell counts were performed using hybrid cell count software. Original magnification, ×100. *H&E* hematoxylin and eosin, *IHC* immunohistochemistry, *FFPE* formalin-fixed paraffin-embedded, *ESCC* esophageal squamous cell carcinoma, *TILs* tumor-infiltrating lymphocytes, *PD-L1* programmed death-ligand 1, *PD-1* programmed death-1, *FOXP3* forkhead box P3, *CD8* cluster of differentiation 8

Previous research has demonstrated that *Bifidobacterium* can enhance anti-tumor immune responses in mouse models;^24^ however, the impact of *Bifidobacterium* on TIL distribution and immune response in ESCC patients remains poorly understood. We hypothesized that *Bifidobacterium* may be a crucial factor influencing the upregulation of anti-tumor immune responses and TIL distribution levels. Therefore, we conducted H&E staining to assess TIL distribution levels, and subsequently evaluated the protein expression of FOXP3, CD8, PD-1, and PD-L1 in ESCC patients by immunohistochemistry (IHC). Representative images of stromal TIL scores are provided in Fig. 3a. Lymphocyte infiltration was observed within the tumors, in the stroma surrounding the tumor, and at the tumor edges. Of the total 213 cases, 198 were classified into three semi-quantitative categories based on TIL levels: score 1 (22%, 43/198), score 2 (52%, 102/198), and score 3 (27%, 53/198). Our analysis revealed that patients with *Bifidobacterium* had significantly lower levels of TILs in tumor tissue (*p* = 0.02), while no significant differences were observed between *Bifidobacterium* and TILs in peritumor tissue (*p* = 0.17).

**Fig. 4:**
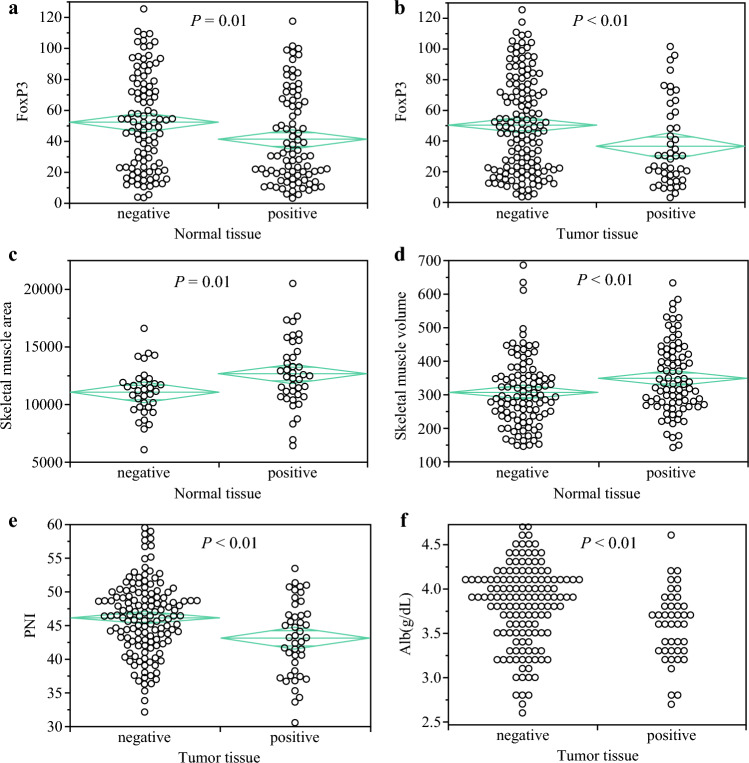
Differences in anti-immune responses and nutritional status based on the presence of *Bifidobacterium* in normal and tumor tissue. **a** Relationship between FOXP3+ T-cell status and *Bifidobacterium* in normal tissue. **b** Relationship between FOXP3+ T-cell status and *Bifidobacterium* in tumor tissue. Relationship between **c** skeletal muscle area and **d** skeletal muscle volume and *Bifidobacterium* in normal tissue. **e** Relationship between PNI and *Bifidobacterium* in tumor tissue. **f** Relationship between albumin and *Bifidobacterium* in tumor tissue. *FOXP3* forkhead box P3, *PNI* prognostic nutrition index, *Alb* albumin

To evaluate the impact of *Bifidobacterium* on anti-tumor immunity, we assessed the association between the existence of *Bifidobacterium* and tumor-infiltrated lymphocyte levels in ESCC tissues. Representative images of immunostaining are shown in Fig. 3b. PD-1 immunohistochemical staining showed no significant difference between *Bifidobacterium*-positive and -negative groups in tumor tissue (*p *= 0.45) [ESM Fig. S1b] or normal tissue (*p *= 0.44) [ESM Fig. S1c]. Among these cases, 25% of tumors were *Bifidobacterium*-positive and 75% were *Bifidobacterium*-negative. Similar trends were observed for CD8 staining. Immunohistochemical staining of 194 cases for CD8 revealed no significant difference in the *Bifidobacterium*-positive and -negative groups in both tumor tissue (*p *= 0.56) [ESM Fig. S1d] and normal tissue (*p *= 0.82) [ESM Fig. S1e]. In tumor and normal tissue, 24% and 47% were *Bifidobacterium*-positive, respectively. FOXP3 expression decreased significantly when *Bifidobacterium* was positive, both in normal tissue (*p* = 0.01) [Fig. 4a] and tumor tissue (*p *< 0.01) [Fig. 4b]. Among these cases, 25% and 48% were *Bifidobacterium*-positive in tumor tissue and normal tissue, respectively. PD-L1 expression showed no significant difference between the *Bifidobacterium*-positive and -negative groups in tumor tissue (*p* = 0.83) and normal tissue (*p *= 0.30). Our analysis shows that the presence of *Bifidobacterium* in normal tissue does not have a statistically significant impact on the quantity of white blood cells (WBCs; *p *= 0.25) [ESM Fig. S1f]. Similarly, no significant correlation was observed between *Bifidobacterium* levels and WBC counts in tumor tissues (*p *= 0.51) [ESM Fig. S1g]. In tumor tissue, 24% were *Bifidobacterium*-positive, while in normal tissue, 47% were positive. These findings suggest that *Bifidobacterium* may enhance anti-tumor immune response by reducing FOXP3^+^ lymphocyte levels.

### Preoperative Nutritional Status

Sarcopenia is consistently associated with a poorer prognosis, highlighting the importance of understanding the relationship between *Bifidobacterium* and preoperative nutritional status. We examined the correlation between the presence of *Bifidobacterium* and various parameters, including skeletal muscle mass, waist circumference, visceral fat, subcutaneous fat, and PNI. Our analysis revealed no significant differences between *Bifidobacterium* and waist circumference, visceral fat, or subcutaneous fat; however, patients with *Bifidobacterium* exhibited a significantly higher skeletal muscle area in normal tissue (*p* = 0.01) [Fig. 4c], although no significant differences were observed in tumor tissue (*p *= 0.08). Similarly, skeletal muscle volume was significantly higher in *Bifidobacterium*-positive patients compared with *Bifidobacterium*-negative patients in peritumor tissue (*p *< 0.01) [Fig. 4d], with no significant difference observed in tumor tissue (*p *= 0.65). Additionally, albumin and PNI levels were lower in the *Bifidobacterium*-positive group than the *Bifidobacterium*-negative group in tumor tissue (*p *< 0.01) [Figs. 4e, f], with no significant difference noted in peritumor tissue (*p *= 0.24).

## Discussion

The immune cells within the tumor microenvironment are crucial prognostic factors that influence the effectiveness of immunotherapy in esophageal cancer.^25–27^ Emerging evidence suggests that *Bifidobacterium* may play an immunomodulatory role.^24,28^ In the meantime, the microbiome has also shown potential cross-talk with skeletal muscle, termed the ‘gut-muscle axis’,^29^ but with limited clinical evidence. Sarcopenia, characterized by muscle loss, has been recognized as a poor outcome in patients with esophageal cancer.^30^ Thus, it is important to investigate the effects of *Bifidobacterium* on anti-tumor immunity and nutritional status in ESCC patients.

In this study, we observed that the presence of *Bifidobacterium* in both tumor tissue and adjacent normal tissues in patients with esophageal cancer had no effect on tumor-infiltrating CD8^+^ T cells but was associated with a decrease in FOXP3^+^ T cells. This finding contrasts with previous research. Studies in mouse models of several cancer types indicate that *Bifidobacterium* can increase the induction and infiltration of tumor-specific CD8^+^ T cells and decrease tumor necrosis factor (TNF)-α levels in tumor tissue, which may inhibit tumor infiltration and induce CD8^+^ T-cell apoptosis.^24,29^ Only one placebo-controlled trial focusing on clinical patients with colorectal cancer showed that *Bifidobacterium* decreased blood TNFα levels without evaluating tumor-infiltrating CD8^+^ T cells.^31^ In contrast to our findings on FOXP3, research has demonstrated that *Bifidobacterium* can increase FOXP3^+^ T-cell levels in mouse models of several cancer types and promote the formation of functional FOXP3^+^ T cells.^32–35^ Our study is the first to evaluate the relationship between *Bifidobacterium* and levels of CD8^+^ and FOXP3^+^ T cells in ESCC patients. One possible explanation for the differences between our results and previous research could be due to the different study subjects used. Furthermore, it is also plausible that multiple specific bacteria contribute to improved anti-tumor immunity in patients.^36^ Another potential reason why patients with *Bifidobacterium* show no significant difference in CD8^+^ T cells may be that those with tumor-infiltrating CD8^+^ T cells may possess more bacteria-reactive CD8^+^ T cells.^37^

Our results indicate that the presence of *Bifidobacterium* in normal tissue correlates with better skeletal muscle area and volume, with similar findings also being reported in other studies. A meta-analysis showed a significant improvement in muscle mass associated with *Bifidobacterium* (95% confidence interval 0.22–0.73; *p* = 0.04).^38^ Studies using mouse models have demonstrated that orally administered *B. longum* for 12 weeks increases grip strength in a dose-dependent manner (*p* < 0.01),^39^ while heat-killed *B. breve* administered orally for 4 weeks increases soleus mass and grip strength.^40^ Recent studies also suggest that B. longum is a potential biomarker that is significantly reduced in sarcopenia patients.^41,42^ These results collectively suggest a potential role for *Bifidobacterium* in enhancing muscle mass and function, but no studies can rule out the possibility that better muscle status can facilitate *Bifidobacterium* colonization. On the other hand, our study found no significant differences between *Bifidobacterium* and waist circumference, visceral fat, or subcutaneous fat, but observed poorer PNI and albumin levels. In contrast, a placebo-controlled trial with 80 pre-obese participants treated with *B. breve* capsules for 12 weeks reported lower fat mass, percentage body fat, visceral fat area, and waist circumference in the *B. breve* group compared with placebo, although blood parameters, including WBCs and albumin, showed no significant changes.^43^ Similarly, a study involving 137 healthy adults treated with *B. lactis* in fermented milk for 12 weeks also demonstrated a reduction in visceral fat.^44^ Discrepancies between our findings and previous studies may stem from nutritional indicators influenced by a combination of bacteria, which our study did not control for. The difference between our results and previous research may also be due to the different study subjects used; previous studies focused on healthy or pre-obese individuals, while our research focused on cancer patients. Patient lifestyle factors could also impact nutritional status, which was also not included in our research. Further studies are warranted to elucidate the relationship between *Bifidobacterium* and nutritional status in esophageal cancer patients.

The current study has some limitations due to its retrospective design, which cannot rule out the possibility of reverse causation. It remains plausible that peritumoral lymphocytes may influence the abundance of *Bifidobacterium* in the tumor environment. Nevertheless, our hypothesis is supported by experimental evidence suggesting a positive impact of *Bifidobacterium* on T-cell activity. However, experimental systems may not fully replicate the complexity of human tumors, the microbiota, and the immune system. The study is further limited by the lack of data on patients’ lifestyle habits and the inability to control for the effects of other bacteria, as in a controlled experiment. Analysis of a large population of human cancer tissues is crucial for understanding the relationship between the microbiota and immunity in cancer. The precise mechanisms underlying the correlation between *Bifidobacterium* and the immune response to cancer cells during tumorigenesis and cancer progression are currently unknown. Subsequent studies are necessary to confirm our findings and explore the potential mechanisms underlying the relationship between *Bifidobacterium* and the immune response.

## Conclusion

Our findings suggest a potential association between the presence of *Bifidobacterium*, improvement of anti-tumor immune responses, and enhancement of preoperative nutritional status in patients with ESCC. These results pave the way for future research aimed at investigating the interactive dynamics between intratumor microbiota and host immunity in the progression of esophageal cancer. Validating these findings could provide valuable insights for developing strategies targeting the gut microbiota and immune cells to prevent and treat esophageal cancer.

## Supplementary Information

Below is the link to the electronic supplementary material.

Supplementary file1 (TIF 630 kb)

## Data Availability

The datasets generated and/or analyzed during the current study are available from the corresponding author on reasonable request.

## References

[1] Bray F, Ferlay J, Soerjomataram I, Siegel RL, Torre LA, Jemal A. Global cancer statistics 2018: GLOBOCAN estimates of incidence and mortality worldwide for 36 cancers in 185 countries. *CA Cancer J Clin*. 2018;68(6):394–424. 10.3322/caac.21492. 30207593 10.3322/caac.21492

[2] Lin Y, Totsuka Y, He Y, et al. Epidemiology of esophageal cancer in Japan and China. *J Epidemiol*. 2013;23(4):233–42. 10.2188/jea.je20120162. 23629646 10.2188/jea.JE20120162 PMC3709543

[3] Abdo J, Agrawal DK, Mittal SK. Basis for molecular diagnostics and immunotherapy for esophageal cancer. *Expert Rev Anticancer Ther*. 2017;17(1):33–45. 10.1080/14737140.2017.1260449. 27838937 10.1080/14737140.2017.1260449 PMC5542819

[4] Pennathur A, Farkas A, Krasinskas AM, et al. Esophagectomy for T1 esophageal cancer: outcomes in 100 patients and implications for endoscopic therapy. *Ann Thorac Surg*. 2009;87(4):1048-1054; discussion 1054-1055. 10.1016/j.athoracsur.2008.12.060 10.1016/j.athoracsur.2008.12.060 PMC2912110 19324126

[5] Overholt BF, Wang KK, Burdick JS, et al. Five-year efficacy and safety of photodynamic therapy with Photofrin in Barrett’s high-grade dysplasia. *Gastrointest Endosc*. 2007;66(3):460–8. 10.1016/j.gie.2006.12.037. 17643436 10.1016/j.gie.2006.12.037

[6] Mönig S, Chevallay M, Niclauss N, et al. Early esophageal cancer: the significance of surgery, endoscopy, and chemoradiation. *Ann N Y Acad Sci*. 2018;1434(1):115–23. 10.1111/nyas.13955. 30138532 10.1111/nyas.13955

[7] Shah MA, Kennedy EB, Catenacci DV, et al. Treatment of locally advanced esophageal carcinoma: ASCO guideline. *J Clin Oncol*. 2020;38(23):2677–94. 10.1200/JCO.20.00866. 32568633 10.1200/JCO.20.00866

[8] Bagchi S, Yuan R, Engleman EG. Immune checkpoint inhibitors for the treatment of cancer: clinical impact and mechanisms of response and resistance. *Annu Rev Pathol*. 2021;16:223–49. 10.1146/annurev-pathol-042020-042741. 33197221 10.1146/annurev-pathol-042020-042741

[9] Schizas D, Charalampakis N, Kole C, et al. Immunotherapy for esophageal cancer: a 2019 update. *Immunotherapy*. 2020;12(3):203–18. 10.2217/imt-2019-0153. 32208794 10.2217/imt-2019-0153

[10] Dzutsev A, Badger JH, Perez-Chanona E, et al. Microbes and cancer. *Annu Rev Immunol*. 2017;35:199–228. 10.1146/annurev-immunol-051116-052133. 28142322 10.1146/annurev-immunol-051116-052133

[11] Jobin C. Precision medicine using microbiota. *Science*. 2018;359(6371):32–4. 10.1126/science.aar2946. 29302001 10.1126/science.aar2946

[12] Nowicki TS, Hu-Lieskovan S, Ribas A. Mechanisms of resistance to PD-1 and PD-L1 blockade. *Cancer J Sudbury Mass*. 2018;24(1):47–53. 10.1097/PPO.0000000000000303. 10.1097/PPO.0000000000000303 PMC5785093 29360728

[13] Z Z, G C, D AD, et al. Insights Into the Oral Microbiome and Barrett’s Esophagus Early Detection: A Narrative Review. *Clin Transl Gastroenterol*. 2021;12(9). 10.14309/ctg.0000000000000390 10.14309/ctg.0000000000000390 PMC8397287 34446641

[14] Li Y, Li Q, Yuan R, Wang Y, Guo C, Wang L. Bifidobacterium breve-derived indole-3-lactic acid ameliorates colitis-associated tumorigenesis by directing the differentiation of immature colonic macrophages. *Theranostics*. 2024;14(7):2719–35. 10.7150/thno.92350. 38773969 10.7150/thno.92350 PMC11103503

[15] Dong Y, Liao W, Tang J, Fei T, Gai Z, Han M. Bifidobacterium BLa80 mitigates colitis by altering gut microbiota and alleviating inflammation. *AMB Express*. 2022;12(1):67. 10.1186/s13568-022-01411-z. 35670877 10.1186/s13568-022-01411-z PMC9174416

[16] Chen S, Fan L, Lin Y, et al. Bifidobacterium adolescentis orchestrates CD143+ cancer-associated fibroblasts to suppress colorectal tumorigenesis by Wnt signaling-regulated GAS1. *Cancer Commun Lond Engl*. 2023;43(9):1027–47. 10.1002/cac2.12469. 10.1002/cac2.12469 PMC10508156 37533188

[17] Lin Y, Fan L, Qi Y, et al. Bifidobacterium adolescentis induces Decorin+ macrophages via TLR2 to suppress colorectal carcinogenesis. *J Exp Clin Cancer Res CR*. 2023;42(1):172. 10.1186/s13046-023-02746-6. 37464382 10.1186/s13046-023-02746-6 PMC10353206

[18] Kim S, Lee HH, Choi W, Kang CH, Kim GH, Cho H. Anti-Tumor Effect of Heat-Killed Bifidobacterium bifidum on Human Gastric Cancer through Akt-p53-Dependent Mitochondrial Apoptosis in Xenograft Models. *Int J Mol Sci*. 2022;23(17):9788. 10.3390/ijms23179788. 36077182 10.3390/ijms23179788 PMC9456556

[19] Mima K, Sakamoto Y, Kosumi K, et al. Mucosal cancer-associated microbes and anastomotic leakage after resection of colorectal carcinoma. *Surg Oncol*. 2020;32:63–8. 10.1016/j.suronc.2019.11.005. 31765952 10.1016/j.suronc.2019.11.005 PMC6986978

[20] Mima K, et al. Fusobacterium nucleatum in colorectal carcinoma tissue and patient prognosis. *Gut*. 2016;65:1973–80. 26311717 10.1136/gutjnl-2015-310101 PMC4769120

[21] Yamamura K, et al. Human microbiome fusobacterium nucleatum in esophageal cancer tissue is associated with prognosis. *Clin Cancer Res*. 2016;22:5574–81. 27769987 10.1158/1078-0432.CCR-16-1786

[22] Yamamura K, et al. Intratumoral fusobacterium nucleatum levels predict therapeutic response to neoadjuvant chemotherapy in esophageal squamous cell carcinoma. *Clin Cancer Res*. 2019;25:6170–9. 31358543 10.1158/1078-0432.CCR-19-0318 PMC6801075

[23] Kosumi K, et al. Intratumour Fusobacterium nucleatum and immune response to oesophageal cancer. *Br J Cancer*. 2023;128:1155–65. 36599917 10.1038/s41416-022-02112-x PMC10006219

[24] Sivan A, Corrales L, Hubert N, et al. Commensal Bifidobacterium promotes antitumor immunity and facilitates anti-PD-L1 efficacy. *Science*. 2015;350(6264):1084–9. 10.1126/science.aac4255. 26541606 10.1126/science.aac4255 PMC4873287

[25] Zheng X, Song X, Shao Y, et al. Prognostic role of tumor-infiltrating lymphocytes in esophagus cancer: a meta-analysis. *Cell Physiol Biochem*. 2018;45(2):720–32. 10.1159/000487164. 29414812 10.1159/000487164

[26] Yagi T, Baba Y, Ishimoto T, et al. PD-L1 expression, tumor-infiltrating lymphocytes, and clinical outcome in patients with surgically resected esophageal cancer. *Ann Surg*. 2019;269(3):471–8. 10.1097/SLA.0000000000002616. 29206673 10.1097/SLA.0000000000002616

[27] Stein AV, Dislich B, Blank A, et al. High intratumoural but not peritumoural inflammatory host response is associated with better prognosis in primary resected oesophageal adenocarcinomas. *Pathology (Phila)*. 2017;49(1):30–7. 10.1016/j.pathol.2016.10.005. 10.1016/j.pathol.2016.10.005 27916317

[28] Mager LF, Burkhard R, Pett N, et al. Microbiome-derived inosine modulates response to checkpoint inhibitor immunotherapy. *Science*. 2020;369(6510):1481–9. 10.1126/science.abc3421. 32792462 10.1126/science.abc3421

[29] Lee SH, Cho SY, Yoon Y, et al. Bifidobacterium bifidum strains synergize with immune checkpoint inhibitors to reduce tumour burden in mice. *Nat Microbiol*. 2021;6(3):277–88. 10.1038/s41564-020-00831-6. 33432149 10.1038/s41564-020-00831-6

[30] Jogiat UM, Sasewich H, Turner SR, et al. Sarcopenia determined by skeletal muscle index predicts overall survival, disease-free survival, and postoperative complications in resectable esophageal cancer: a systematic review and meta-analysis. *Ann Surg*. 2022;276(5):e311–8. 10.1097/SLA.0000000000005452. 35794004 10.1097/SLA.0000000000005452

[31] Zaharuddin L, Mokhtar NM, Muhammad Nawawi KN, Raja Ali RA. A randomized double-blind placebo-controlled trial of probiotics in post-surgical colorectal cancer. *BMC Gastroenterol*. 2019;19(1):131. 10.1186/s12876-019-1047-4. 31340751 10.1186/s12876-019-1047-4 PMC6657028

[32] Mi H, Dong Y, Zhang B, et al. Bifidobacterium infantis ameliorates chemotherapy-induced intestinal mucositis via regulating T cell immunity in colorectal cancer rats. *Cell Physiol Biochem*. 2017;42(6):2330–41. 10.1159/000480005. 28848081 10.1159/000480005

[33] Feleszko W, Jaworska J, Rha RD, et al. Probiotic-induced suppression of allergic sensitization and airway inflammation is associated with an increase of T regulatory-dependent mechanisms in a murine model of asthma. *Clin Exp Allergy*. 2007;37(4):498–505. 10.1111/j.1365-2222.2006.02629.x. 17430345 10.1111/j.1365-2222.2006.02629.x

[34] O’Mahony C, Scully P, O’Mahony D, et al. Commensal-induced regulatory T cells mediate protection against pathogen-stimulated NF-kappaB activation. *PLoS Pathog*. 2008;4(8):e1000112. 10.1371/journal.ppat.1000112. 18670628 10.1371/journal.ppat.1000112 PMC2474968

[35] López P, González-Rodríguez I, Gueimonde M, Margolles A, Suárez A. Immune response to Bifidobacterium bifidum strains support Treg/Th17 plasticity. *PloS One*. 2011;6(9):e24776. 10.1371/journal.pone.0024776. 21966367 10.1371/journal.pone.0024776 PMC3178565

[36] Matson V, Fessler J, Bao R, et al. The commensal microbiome is associated with anti–PD-1 efficacy in metastatic melanoma patients. *Science*. 2018;359(6371):104–8. 10.1126/science.aao3290. 29302014 10.1126/science.aao3290 PMC6707353

[37] Rong Y, Dong Z, Hong Z, et al. Reactivity toward Bifidobacterium longum and Enterococcus hirae demonstrate robust CD8+ T cell response and better prognosis in HBV-related hepatocellular carcinoma. *Exp Cell Res*. 2017;358(2):352–9. 10.1016/j.yexcr.2017.07.009. 28694023 10.1016/j.yexcr.2017.07.009

[38] Prokopidis K, Giannos P, Kirwan R, et al. Impact of probiotics on muscle mass, muscle strength and lean mass: a systematic review and meta-analysis of randomized controlled trials. *J Cachexia Sarcopenia Muscle*. 2023;14(1):30–44. 10.1002/jcsm.13132. 36414567 10.1002/jcsm.13132 PMC9891957

[39] Lee MC, Hsu YJ, Chuang HL, et al. In Vivo Ergogenic Properties of the Bifidobacterium longum OLP-01 Isolated from a Weightlifting Gold Medalist. *Nutrients*. 2019;11(9):2003. 10.3390/nu11092003. 31450615 10.3390/nu11092003 PMC6770164

[40] Toda K, Yamauchi Y, Tanaka A, et al. Heat-killed bifidobacterium breve b-3 enhances muscle functions: possible involvement of increases in muscle mass and mitochondrial biogenesis. *Nutrients*. 2020;12(1):219. 10.3390/nu12010219. 31952193 10.3390/nu12010219 PMC7019314

[41] Wang Z, Xu X, Deji Y, et al. Bifidobacterium as a potential biomarker of sarcopenia in elderly women. *Nutrients*. 2023;15(5):1266. 10.3390/nu15051266. 36904265 10.3390/nu15051266 PMC10005572

[42] He Y, Cui W, Fang T, Zhang Z, Zeng M. Metabolites of the gut microbiota may serve as precise diagnostic markers for sarcopenia in the elderly. *Front Microbiol*. 2023;14:1301805. 10.3389/fmicb.2023.1301805. 38188577 10.3389/fmicb.2023.1301805 PMC10768011

[43] Minami J, Iwabuchi N, Tanaka M, et al. Effects of Bifidobacterium breve B-3 on body fat reductions in pre-obese adults: a randomized, double-blind, placebo-controlled trial. *Biosci Microbiota Food Health*. 2018;37(3):67–75. 10.12938/bmfh.18-001. 30094122 10.12938/bmfh.18-001 PMC6081611

[44] Takahashi S, Anzawa D, Takami K, et al. Effect of Bifidobacterium animalis ssp. lactis GCL2505 on visceral fat accumulation in healthy Japanese adults: a randomized controlled trial. *Biosci Microbiota Food Health*. 2016;35(4):163–71. 10.12938/bmfh.2016-002. 27867803 10.12938/bmfh.2016-002 PMC5107634

