# Supplementary figures and images for: The Relationship Between Gut Microbiome Bifidobacterium and Anti-tumor Immune Responses in Esophageal Squamous Cell Carcinoma
Source: Ann Surg Oncol. 2025 Mar 4;32(5):3828–38. doi: 10.1245/s10434-024-16288-4 (PMC11976794; doi:10.1245/s10434-024-16288-4)

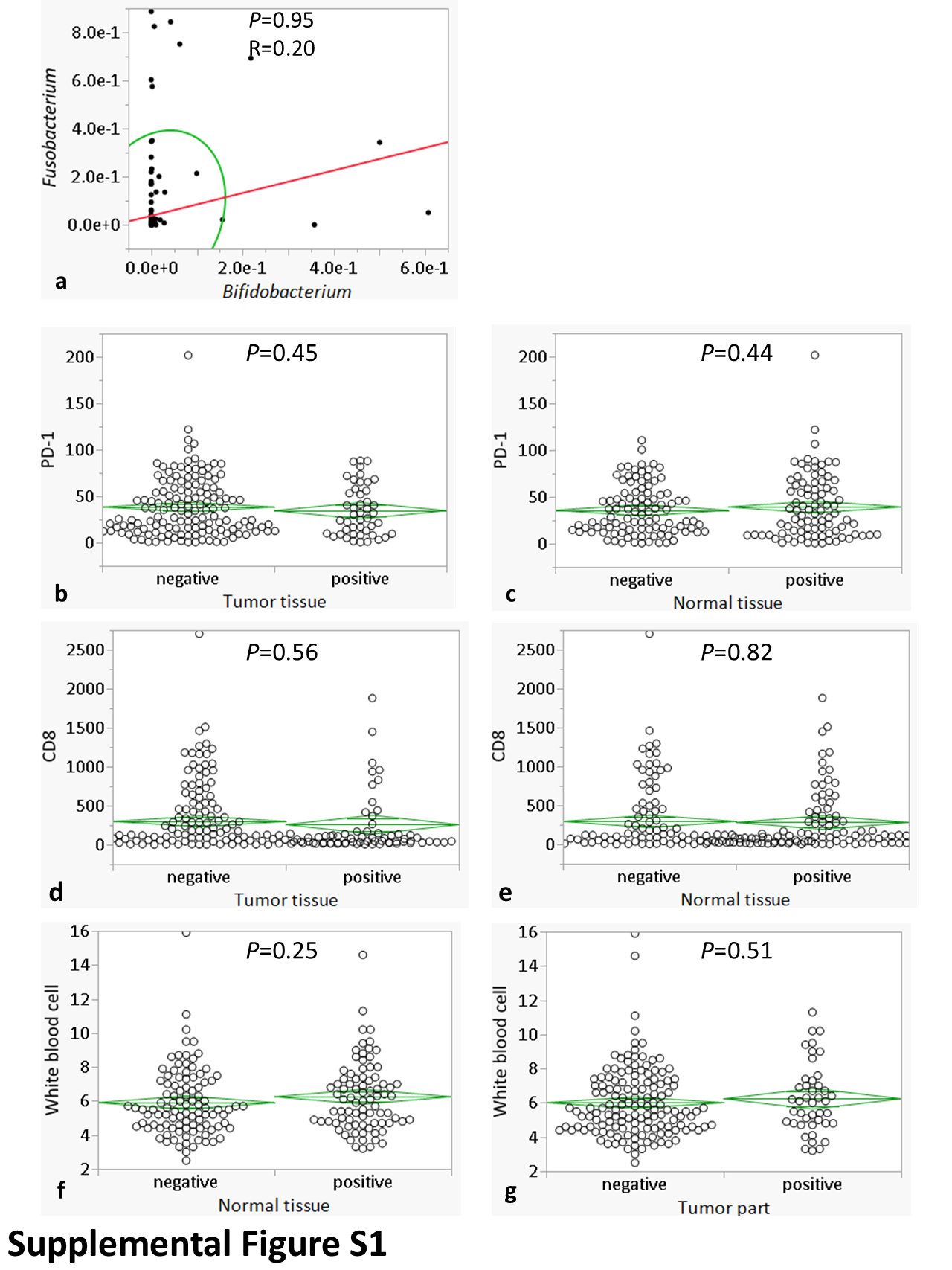

Supplement: Supplementary file 1 — Supplementary file1 (TIF 630 kb) [file 10434_2024_16288_MOESM1_ESM.tif]
